# Innovative Simulation Strategies in Education

**DOI:** 10.1155/2012/765212

**Published:** 2012-04-05

**Authors:** Michelle Aebersold, Dana Tschannen, Melissa Bathish

**Affiliations:** Nursing Business and Health Systems, University of Michigan School of Nursing, 400 North Ingalls, Ann Arbor, MI 48109, USA

## Abstract

The use of simulation in the undergraduate nursing curriculum is gaining popularity and is becoming a foundation of many nursing programs. The purpose of this paper is to highlight a new simulation teaching strategy, virtual reality (VR) simulation, which capitalizes on the technological skills of the new generation student. This small-scale pilot study focused on improving interpersonal skills in senior level nursing students using VR simulation. In this study, a repeated-measure design was used to evaluate the effectiveness of VR simulation on improving student's performance over a series of two VR scenarios. Using the Emergency Medicine Crisis Resource Management (EMCRM) tool, student performance was evaluated. Overall, the total EMCRM score improved but not significantly. The subscale areas of communication (*P* = .047, 95% CI: − 1.06, −.007) and professional behavior (*P* = .003, 95% CI: − 1.12, −.303) did show a significant improvement between the two scenario exposures. Findings from this study show the potential for virtual reality simulations to have an impact on nursing student performance.

## 1. Introduction

The use of simulation in health care education is gaining popularity and is becoming a foundation for many undergraduate nursing programs. Most of the studies in the area of medical simulation focus on high-fidelity simulators or task trainers. However, there are potential correlations between the effectiveness of mannequin-based simulators and other types of simulation including virtual reality simulation. According to Gaba [1], simulation is a “technique” not a technology and focuses on recreating real-life situations to allow students to practice or gain skills in a safe environment. Many simulation centers use a variety of simulation techniques including low-fidelity task trainers such as IV insertion arms and high-fidelity human patient simulators, such as Laerdal's SimMan. High-fidelity mannequin-based simulation has been proven to be effective in both knowledge and skill acquisition and transfer [2–5]. A recent systematic review by Lapkin et al. [6] found that simulation improves critical thinking skills, knowledge acquisition, and the ability to identify a deteriorating patient. Another systematic review by Harder [7] found the use of simulation, when compared to other teaching methods, and improved health care students skills in the majority of studies examined.

Human patient simulators are beneficial for working with students in many clinical patient situations; however, other methods of simulation, such as simulations in virtual environments, may be appropriate for certain settings and learner objectives and can be used in addition to mannequin-based simulation. The advantage to using other methods of simulation such as virtual simulation may increase student exposure to simulation in areas where access to a simulation center is limited. The ability to facilitate active learning in multiple venues increases the opportunities for students to gain experiential learning critical to their success. The current generation of students is exposed to more computer based learning techniques than previous generations and social networking is a common way students engage with each other both in and out of the classroom. These computer savvy students or digital natives, people who were born into and raised in the digital world [8], are likely to be comfortable engaging in virtual simulations, therefore making this a viable simulation technique.

The purpose of this paper is to highlight a virtual simulation technique that capitalizes on the technological skills of this new generation of students with the purpose of developing key interpersonal skills (e.g., communication, delegation, conflict management, decision making, etc.) critical to the success of the new graduate nurse in the clinical environment. Increasing the amount and types of simulation exposure could enhance overall student learning and allow for the best utilization of simulation resources.

Education for nursing students can be challenging when only random learning opportunities are available, and clinical experiences are dependent on the patient population or current practice environment. Thus, no assurance of knowledge acquisition of many vital concepts, such as conflict management, empowerment, delegation, ethics, and priority setting can be made. Simulations provide students with an opportunity to practice their skills in a safe environment, allowing for skill refinement with repeated exposure over time.

The use of simulation has increased in many nursing programs and due to this increase in use it may be difficult to schedule all of the simulations necessary to provide students with a comprehensive skill set upon graduation. One avenue for overcoming these barriers is the use of a virtual environment or virtual world as a representative training area for students to engage in simulations that focus on interpersonal skills such as communication or critical thinking skills. A virtual world is a “computer based, simulated multi-media environment” ([9], Page 233). A virtual world is typically set up to run over the World Wide Web wherein users create and identify themselves through an avatar, an online manifestation of self.

Some virtual worlds are called multiuser virtual environments (MUVEs) because they allow for more than one user to be in the environment and interact with other users in a synchronous fashion. The most popular and mature MUVE that is currently being used in education is Second Life [10]. Second Life (SL) is an online open-access MUVE developed and maintained by Linden Labs. SL allows anyone to open an account, set up a personalized avatar, and download their program for free. The technical requirements to run the program are found on many computers today. SL allows students to participate in real-life situations with other students in a MUVE through the use of avatars while receiving simultaneous interactive prompting and instruction by facilitators. Students have the ability to participate in any location where they can access SL via the internet. The use of avatars allows students a feeling of being “physically” present in the SL environment, allowing training to be in a safe, controlled setting where students may practice and enhance their skills [11].

SL has been used as an educational platform for many different medical, health, and nursing skills, such as identifying certain heart sounds, assessing patients, and engaging in reflective practice [9]. It allows students to gain experience in real-life situations in an environment that can be facilitated or set up by the educator. Students are able to gain the appropriate skills and make clinical decisions based on their learning while avoiding mishaps in patient safety that could occur in an actual clinical area. In a virtual learning environment, no harm is done to patients if an incorrect procedure or medication is administered. Unfortunately to date, there are few studies that examine the effectiveness of MUVE's such as SL on student knowledge and/or skill acquisition. This is due in part to the newness of the technology and the challenges in studying the environment. A brief summary of the few studies found in the literature as follows.

In one study, the use of a virtual learning environment led to better reflection between online students, which may support the creation of communities of practice [9]. In another study, paramedic students using SL for problem-based learning, found the environment more authentic and collaborative than using paper-based problem solving scenarios. The researchers also found the SL environment allowed for feedback to the students from their virtual “patients” which increased the benefit of the learning environment for the learners [12].

Commercial and educational institutions are using SL or other MUVE's to assist in their training/educational programs and curriculum. MICHELIN automotive tires developed a training environment in SL to train their information systems (IS) personnel in the United States, Europe, and Asia to ensure global alignment of processes and IS solutions [13]. The University of Kansas Medical Center uses SL to run training simulations for anesthesia induction in a setting that replicates their current operation room [13]. There are many uses for SL, but very few studies are published yet.

One study did focus on comparing the outcomes of virtual reality (VR) training to mannequin-based simulation. In this study [14], subjects were assigned to either a mannequin-based simulation or a VR simulation for team training in the emergency department. The results indicated both groups showed significant improvement in performance after completing the training. Simulation can be an effective education and training method educators and faculty can use to facilitate student learning, particularly in the health professions where certain critical skills are necessary for optimal and safe patient care. Therefore, measuring the ability of simulation, in particular virtual simulation, to improve learner skills is of vital importance.

## 2. Conceptual Framework

The conceptual framework used to guide this research is based on Ericsson's [15] work on expertise. Ericsson's framework posits that to acquire expert performance one must engage in deliberate practice activities that are clearly focused on improving some aspect of performance. Most students tend to improve performance with experience; however, Ericsson's theory states that experts are those individuals who continue to improve beyond the level needed to perform adequately and become recognized as experts in their domain. Although students are just beginning in their skill development the underpinnings of Ericsson's theory can help educators focus deliberate efforts to improve selected skills or tasks. During this process, students are instructed to improve certain aspects of their performance for a well-defined task, such as communicating to a health care provider regarding a patient's status. The student is then given immediate detailed feedback on their performance which they can reflect upon and continue to practice during subsequent training sessions. Simulation is one of the techniques that can be used to engage professionals in deliberate practice of skills and has the ability to improve performance in professionals that require deliberate, goal-oriented, and structured practice [14].

## 3. Materials and Methods

The specific aim of this study was to examine the relationship between student learning and use of virtual simulated clinical experiences. A repeated-measure design was used to evaluate the effectiveness of virtual simulation on improving student's performance over a series of two virtual simulation scenarios. This study was conducted over one academic semester with a convenience sample of senior nursing students at one midwestern, university-based School of Nursing. The students were traditional undergraduate students taking Leadership and Management. Student demographic information was not collected; however, the majority of students were young, post high school students completing their first degree. At total of 61 students participated in this study. They received education in the traditional manner (i.e., lecture and seminar discussion) in addition to the two virtual simulations. Institutional Review Board review was obtained, and the study was considered exempt.

### 3.1. Instruments

Student (avatar) performance in the virtual scenarios was measured using 8 of the 10 items from the Emergency Medicine Crisis Resource Management (EMCRM) tool, developed by Youngblood et al. [14]. This tool was developed to evaluate participant's crisis management skills in a virtual emergency department. The EMCRM assesses subject's team leadership skills, including utilization of information and resources and overall ability to communicate and facilitate task completion. Initial reliability testing of the tool was supported with a Cronbach's alpha of 0.96 [14]. The tool was reduced by two items (those items did not apply to the virtual simulations designed for this study) to measure nurse's performance in a crisis situation in an acute care unit setting. The remaining categories included leadership, communication, delegation, attention, information utilization, resource utilization, early call for help, and professional behavior. The definition of these categories was adjusted to account for nursing student expectations of behavior. Cronbach's alpha on the revised EMCRM was 0.9 as measured during this study.

Student satisfaction was measured through a satisfaction questionnaire developed for the initial beta testing of the SL environment [16]. The six-question survey measured student's perceptions on a 5-point Likert scale (strongly disagree to strongly agree). Questions included SL experiences helped prepare me for clinical, SL experiences resembled a realistic clinical environment, SL experiences reinforced the course objectives, SL experiences were an effective learning experience, SL experiences improved my decision making skills, and overall the SL experiences were a positive experience. Four open-ended questions were also asked: what I like most about the experience in SL, what I liked least about the experience in SL, and how do you rate this experience in comparison with other simulations (i.e., SimMan) and suggestions for improvement.

### 3.2. Intervention

**Figure 1:**
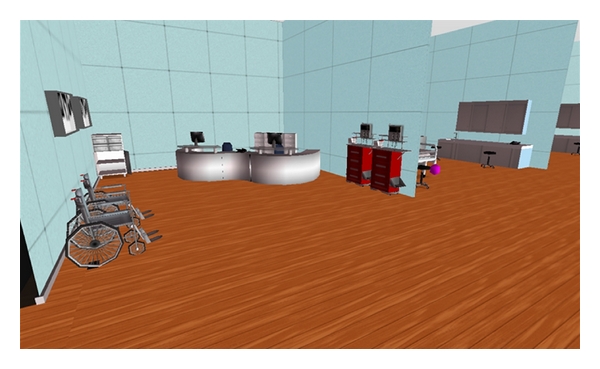
Second life nursing station.

**Figure 2:**
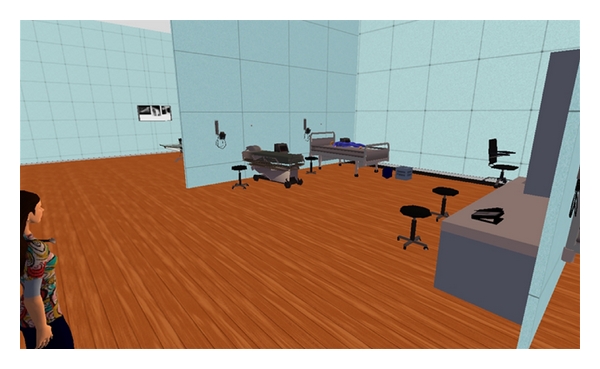
Second life patient care room.

 The intervention consisted of two virtual scenarios that the students participated in at two different times during one academic semester. Prior to creating the simulations, a hospital unit was developed on the university owned space in SL, which was subsequently used as a training environment for the virtual simulation scenarios [16]. The virtual unit consists of eight acute care beds with mannequins (patients), a nursing conference room, a nursing station, and a variety of medical equipment staged throughout the unit to enhance realism of the unit (Figures 1 and 2). The unit was constructed for minimal cost as the University already owned the space and had developed the building used. The costs included buying items for the unit (e.g., hospital beds, desks, equipment, etc.) and some developer time to put up walls and create or modify objects used on the inside of the building. The total project cost was under $3000. The University provided in-house experts to the project team to support the training and development of the facilitators using SL for this project.

Two scenarios were developed by two content and simulation experts. Objectives for the scenarios were developed in alignment with the Quality and Safety Education for Nurses (QSEN) recommendations (Cronenwett et al. [17]) and The Baccalaureate Essentials for Practice [18], in addition to specific course and overall nursing program objectives. Specifically, objectives were focused on QSEN's teamwork, collaboration and patient safety objectives and the Essentials IV and VIII focusing on interprofessional communication and professionalism.

As described in Table 1, Scenario 1 involved a safety issue and a medication adverse event, and Scenario 2 involved a difficult interprofessional communication situation. Each scenario required the students to use a similar skill set (patient safety, leadership, communication with healthcare providers, feedback to colleagues, appropriate use of resources and followup, and delegation). The scenarios were developed and beta tested using second career nursing students and faculty prior to use in this study [16]. Students in this study were given an overview of SL during the didactic portion of their fall course. Each student was also given a Power Point handout that described the basics of SL such as how to set up an account, create an avatar, move your avatar, and use the chat function. Students were encouraged to develop their own avatars and explore the world prior to the simulations. For each simulation, students who played a role in the scenario (e.g., role of RN, unlicensed assistive personnel, patient) went to the school computer lab and logged into SL, while the remaining students were in another classroom watching the virtual interaction via LCD projector through one of the facilitator's avatars. The students used avatars that were already created and were present in the training environment. The students needed to use the directional keys on the keyboard to move their avatar and a texting function called chat to “speak” to each other. These were the only skills necessary to participate in the virtual simulations.

Prior to each simulation the students who played a role in the virtual environment using an avatar were once again given a brief overview of SL and how to manipulate their avatars. They had an opportunity to practice for a few minutes moving their avatars and using the chat function before the simulation began. A facilitator was present during the entire simulation to answer questions or troubleshoot any problems the students had during the simulation.

Five groups of approximately 15 students participated in the two scenarios at two different points in time (in Scenario 2 two of the five groups participated at the same time). It is important to note that not all students were actually able to play a role in the scenarios. Most students were observers watching the simulation scenario. This is consistent with mannequin-based simulation in which students may play roles in the simulation or observe the simulation. All students, however, do participate in the debriefing of the scenario and are exposed to key learning points. Scenario 1 was conducted in September 2009, and Scenario 2 was conducted in November 2009. The students were all given background information on the scenario (i.e., some students playing roles in the simulation were given cue cards if a certain response was required during the simulation), and then the scenario was started. The students who played the roles were volunteers from the larger group. The communication was done using the text chat function in SL so that all students could see the communication interaction. The text chat function in SL is similar to most online chat programs and allows the students to type in their communication and others to respond. The communication stream is visible on the computer to all avatars in the area. One of the facilitators ran the scenarios in SL by giving the students their instructions via text chat and passing note cards (written information that can be passed in-world between avatars) when appropriate. The simulations ran for approximately 10–15 minutes. At the conclusion of the scenario, all of the students (including those who played a role) met in the classroom and were thoroughly debriefed by a trained facilitator. Text chat logs from the simulation were saved for future analysis. Students who did not play a role in the scenario were asked for specific feedback during the debriefing on how they might have handled the situation. All 61 students participated in both virtual simulations; however, the majority of those students participated as observers. Different students played roles in each scenario allowing a larger portion of the 61 students to directly participate. After the completion of the virtual scenarios all students were asked to complete the satisfaction questionnaire.

### 3.3. Data Collection

During the scenario only the charge nurse role and the bedside nurse role were evaluated using the shortened EMCRM by the trained observers. The other roles played by students (tech and family member) and facilitators were part of the scenario flow but not scored. The bedside role was scored in all the scenarios, but the charge role was only scored if the charge nurse was called upon by the bedside nurse to help out which accounts for the different number of observed cases between scenario 1 and 2 (8 versus 7). In other words, there were 8 observations for scenario one and 7 observations for scenario 2 that were analyzed in the study.

Multiple raters independently observed the subjects' performance in key roles (day RN or bedside nurse, charge RN) on both scenarios. The raters were trained by the researchers prior to the virtual simulations in the use of the tool and the behaviors that would indicate the various scores. Each rater was given an overview of the EMCRM tool and the definitions for each item. Examples of what that behavior looked like were discussed. All raters observed the simulation at the same time and scored their sheets. After the initial scenario, raters were able to compare their results and any disagreements in ratings were discussed immediately after the simulation. Raters then continued to score each subsequent simulation independently. The raters used a 5-point Likert scale (1 = poor; 5 = excellent) to assign scores for each of the 8 items on the scale, for a total possible score of 40. These scores were then averaged for a single consensus score for each subscale for each group and role. Scenario 1 had a total of 8 roles (bedside nurse and charge nurse) for five groups, and scenario 2 had 7 roles (bedside nurse and charge nurse) for four groups that were scored. Data was entered into PASW for Windows, Version 17.0. Descriptive statistics for all continuous variables were computed, and independent *t*-tests were performed to compare student performance over time.

## 4. Results and Discussion

As noted in Table 2, overall student performance (total EMCRM score) on the second virtual unit scenario (average score 31.90, SD 3.19) was slightly better than performance on the first scenario (average score = 30.32, SD 4.09) showing some improvement with exposure to this type of computer-based simulation.

Independent *t*-tests were performed to compare the average value of each of the subscale variables between Scenario 1 and Scenario 2 to determine if there were significant differences in performance based on repeated exposure. Team communication (*P* = .047, 95% CI: −1.06, −.007) and professional behavior (*P* = .003, 95% CI: −1.12, −.303) showed a significant difference between the two scenario exposures. Specifically, the student's ability to communicate with the team and their professional behavior improved from scenario 1 to scenario 2.

The satisfaction survey was completed by 61 students. Student satisfaction scores (Table 3) showed that students rated the variables slightly better than neutral on their agreement with the variables. The scores ranged from 1 (strongly disagree) to 5 (strongly agree). Preparation for clinical was rated at a mean 3.14 (SD  .94), realism of the environment was 3.18 M (SD 1.05), and reinforced objectives was 3.55 M (SD  .87). Reinforced objectives was the highest scoring item. Consequently the effectiveness of the learning environment scored the lowest at a mean of 3.07 (SD  .98). Improving decisions scored 3.28 M (SD  .92), and the overall experience was rated 3.32 M (SD  .85).

Student comments were mixed. One student responded, “I could see this sort of thing happening in a real clinical setting. The real-life scenario (i.e., charge nurse scenario) helped me make better decisions….” Another comment, “I found the experiences displayed in second life were realistic and discussing what could have/should have been done was helpful….” Several comments indicated students did not like the text chat function and found it difficult to read when they were observing. “The simulations were very realistic. If there is a way to make the typed font bigger it would be easier to read”. Another comment, “The Second Life is an interesting approach to clinical—but I prefer the real-life sim because I prefer the verbal “out loud” conversation—it is more real-life.”

The EMCRM results show that team communication and professional behavior did show significant improvement. This may have improved in part because the students became more comfortable with the virtual world and the interaction with other members (avatars) in addition to the learning that occurred during scenario 1. Subjects in this study did demonstrate improvement in their team leadership skills (total EMCRM score) from scenario 1 to scenario 2; however, the improvement was not significant. Many of the behaviors evaluated in the virtual scenarios are complex, such as work delegation and attention allocation, and the two scenarios may have not been enough practice to show significant improvement in those areas. It is also important to note that there were a small number of cases in each scenario. Despite the fact that 61 students participated in the virtual simulations, only 8 cases were rated in scenario 1, and 7 cases in scenario 2 were scored because only 2 students could play a role in each of the virtual simulations.

This pilot study demonstrates that participation in VR simulation helps students improve performance; however, more work is needed to determine how significant the VR simulations contributed to this learning. These results are encouraging as further research using pre- and post-tests could further evaluate improvement in performance with repeated exposure, deliberate practice, and familiarity with virtual world learning.

## 5. Limitations

 Several limitations have been identified in this study. One obvious limitation is the study's small sample size which impacts the power of the study, perhaps increasing the chance of a type II error in data analysis. There were a small number of cases for each outcome variable of interest and as such, the bounds of normality may be stretched. The sample for this study was a convenience sample of fourth year nursing students from the same university, making generalizability of results to other populations unknown, and a second study limitation. Interrater reliability was not conducted as the scores of the raters were averaged into a single consensus score for each simulation.

## 6. Conclusions

Virtual world simulation environments offer a unique and potentially cost-effective method of teaching nursing skills related to leadership and management skills in facilities that have access to virtual environments such as SL. The university owned space in SL provides a virtual unit environment that has the potential to offer valuable, practical, real-world experiences for future nursing students. The total cost of the project to set up the virtual hospital was under $3000. This presumes that one has access to a space in SL that is already being maintained, and no additional costs are incurred for space purchase and ongoing costs such as lease fees or information technology fees. The cost would be much higher for educators who do not have access to SL. The cost savings involved would be a savings on lab fees and supplies associated with high-fidelity simulation centers. This type of environment allows for the practice and learning of interpersonal skills such as communication, teamwork, and delegation which are critical to patient safety and improved patient outcomes.

Students are challenged with learning both technical and nontechnical skills to achieve competency in their future roles as practicing nurses. Ericsson's [19] work in the area of deliberate practice and expertise provides a framework for educators to use in assisting nurses and nursing students in achieving these skills. The results of this study demonstrate that improvement can be seen over time as students practice those skills. However, this improvement will need to be reinforced through subsequent simulations, and SL can provide a less expensive training environment that can be used to practice many nontechnical skills such as clinical judgment, teamwork, leadership, and communication. It can also provide an opportunity for nurses to participate in simulations from home or anywhere they can access the internet, providing educators with a new, flexible option for training. In addition, this method of simulation may maximize opportunities for multidisciplinary teams to interact and practice necessary skills for high quality of care. Adding virtual simulations to well-established curriculum that includes mannequin-based simulations can be an added benefit and allow for expanding learning opportunities for students.

## Figures and Tables

**Table 1:** Virtual scenarios.

Scenario	Objectives	Roles	Situation	Expected actions
(1) Medication safety	(1) students will demonstrate the correct response to a medication error; (2) students will use appropriate communication skills when delivering peer feedback; (3) students will demonstrate leadership skills in difficult situations.	(1) Bedside day nurse (2) Charge nurse (3) Bedside night nurse	The scenario starts at the beginning of the shift as the bedside nurse is given instructions to hang an antibiotic ordered for the patient. When he/she arrives at the bedside they find an empty bag of a different antibiotic attached to the patient's intravenous line. The antibiotic hanging was not ordered for the patient, has another patient's name on it and the patient is allergic to the medication. The student is told to proceed as they would if this happened in the clinical environment.	(1) assess the patient for a reaction to the antibiotic and provide any necessary emergency interventions; (2) stop the antibiotic; (3) notify the physician and carry out any orders given; (4) notify the charge nurse; (5) complete an incident report; (6) follow up with the night nurse who hung the antibiotic found hanging at the bedside.

(2) Interprofessional communication	(1) students will recognize inappropriate communication; (2) students will use appropriate conflict management skills; (3) students will use resources appropriately.	(1) Bedside nurse (2) Charge nurse (3) Physicia (played by faculty) (4) Assistive personnel Patient family member	The scenario starts as the student playing the role of the nurse is directed to go to the bedside and begin discharge teaching for a patient who is waiting to go home. A physician comes in and is upset about the care related to a different patient and begins a confrontational verbal dialogue at the bedside. The student is again directed to handle the situation as they would if they were in the clinical environment.	(1) get the physician away from the bedside to continue the conversation; (2) use conflict management skills to resolve the situation; (3) get the charge nurse involved if situation continues; (4) conduct any necessary followup with the situation the physician was upset about.

**Table 2:** Results comparing scenario 1 (time 1) to scenario 2 (time 2).

ECRM	Scenario 1 mean	Scenario 2 mean	Significance (*P* value)
Leadership	4.13	4.22	.643
Team communication	3.79	4.33	.047*
Work delegation	3.64	3.75	.698
Attention allocation	3.92	4.10	.456
Information utilization	3.76	4.25	.085
Resource utilization	3.54	3.42	.783
Early call for help	3.70	3.25	.359
Professional behavior	3.83	4.54	.003*

Total score	30.32	31.90	.424

*Indicates significance *P* < .05.

**Table 3:** Survey responses (*n* = 61).

Variable	*N*	Minimum	Maximum	Mean	Standard Deviation
Preparation for clinical	61	1.00	5.00	3.14	.94
Resemblance of realistic clinical environment	60	1.00	5.00	3.18	1.05
Reinforced course objectives	60	1.00	5.00	3.55	.87
Effective learning experience	61	1.00	5.00	3.07	.98
Improved decision making skills	61	1.00	5.00	3.28	.92
Overall positive experience	61	1.00	5.00	3.32	.85

*Scores range from 1 = strongly disagree to 5 = strongly agree.
